# The most requested factors in clinical skills exams for evaluating novice physicians: an internet-based survey of the general public in Japan

**DOI:** 10.1186/1472-6920-13-74

**Published:** 2013-05-24

**Authors:** Junji Otaki, Shizuko Nagata-Kobayashi, Ayumi Takayashiki, Maiko Ono, Motoharu Fukushi, Shinji Matsumura

**Affiliations:** 1 Center for Medical Education, Graduate School of Medicine, Hokkaido University, North 15, West 7, Kita-ku, Sapporo 060-8638, Japan; 2 Tokyo Medical University, Tokyo, Japan; 3 School of Medicine, University of Tsukuba, Tsukuba, Japan; 4 Karatsu Municipal Hospital, Saga, Japan; 5 Ishibashi Clinic, Tokyo, Japan; 6 Matsumura Family Clinic, Tokyo, Japan

**Keywords:** OSCE, Clinical skills test, National medical licensure examination, Internet-based survey, Opinion of the general public

## Abstract

**Background:**

Clinical skills tests have been added to the national medical licensure examinations in Canada, the U.S., Korea and Switzerland. Adding a clinical skills test to the Japanese national medical licensure examination should also be considered under the Medical Practitioners Act. On the other hand, such tests might be costly and represent an economic burden to the nation’s citizens. Thus, it is appropriate to obtain the opinion of the general public for the introduction of such tests. Although a clinical skills test can measure various competencies, it remains uncertain as to what should be measured. In this study, we aimed to ascertain public opinion regarding the clinical skills demanded of novice physicians.

**Methods:**

We conducted an internet-based survey of the general public in Japan. We randomly selected 7,213 people aged 20 to 69 years. The main topics surveyed included: whether the Japanese government should add a skills test to the existing national medical licensure examination; what kind of skills should be included in this test; and who should pay for the examination.

**Results:**

Of 3,093 (1,531 men and 1,562 women) people who completed the questionnaire (completion rate 42.9%), 90.5% (n = 2,800) responded that a clinical skills test should be part of the national medical licensure examination. The main skills which respondents thought should be included were “explaining and discussing medical issues in an appropriate manner to patients” (n = 2,176, 70.4%), “accurately diagnosing problems by conducting a physical examination” (n = 1,984, 64.1%), and “carefully interviewing patients to make a diagnosis” (n = 1,663; 53.8%). Three-fifths of the respondents (n = 1,900; 61.4%) responded that more than half of the cost of the examination should be paid by the Japanese government.

**Conclusions:**

The majority of respondents indicated that a clinical skills test should be added to the national medical licensure examination. These respondents who represent the general public were requesting the verification of communication, diagnostic interview and diagnostic physical examination skills. Medical educators should incorporate these public requests, and teach and assess medical students accordingly.

## Background

Within the last 20 years, clinical skills tests have been added to the national medical licensure examinations in Canada, the U.S., Korea and Switzerland. Similarly, adding a clinical skills test to the Japanese national medical licensure examination should also be considered to meet demand in order to assure novice physicians’ clinical competence under the Medical Practitioners Act. Japanese government and Japanese Society for Medical Education are considering when and how to start it.

However, such tests might be costly and represent an economic burden to the nation’s aging citizens. Public is a part of stakeholders of medical education, and needs assessment of stakeholders are essential for developing an innovation of it [1]. Thus, it is necessary to obtain the opinion of the general public when deciding the introduction of such tests, similarly to the conduct of a U.S. poll which supported the recent addition of skills tests to the United States Medical Licensing Examination [2]. We had a hypothesis that most Japanese citizens agree to introduce skills test also in Japan.

In addition, although a clinical skills test can measure various competencies such as communication, medical interviewing, physical examination, case presentation, chart writing, and clinical procedures, it remains uncertain what “the public opinion is to what should be measured.” In this study, we aimed to clarify public opinion regarding which competencies a clinical skills test should measure. Some data of this study were previously published [3]. With permission from the publisher, we report whole picture of our study in this article.

## Methods

We conducted an internet-based survey from September 22 to 27, 2006 with help of an internet survey firm which has one of the largest panels. From a panel of 534,759 (264,039 men and 270,720 women) people living in Japan, we randomly selected 7,213 people aged 20 to 69 years who declared neither medical professionals nor medical workers. The instructions of the survey web page indicated that participation in this survey was voluntary and that responses would be kept confidential.

We developed a 6-item questionnaire based on the results of our preceding qualitative study, including 9 individual interviews and 3 focus groups about lay people’s understanding of medical education, [4] a questionnaire survey among 521 patients of 10 Japanese teaching hospitals selected by a cluster sampling, [5] and a previous study about people’s level of skepticism toward medical care and health utilization [6].

The topics of the questionnaire were as follows: 1) what kind of skills were asked for novice physicians; 2) respondents’ levels of skepticism toward medical care and health utilization; 3) whether the Japanese government should add a skills test to the existing national medical licensure examination; 4) who should pay the cost for the examination; 5) respondents’ characteristics; and 6) an open-ended question regarding the important conditions for novice physicians. Respondents’ preferences regarding the clinical skills test were rated on a 5-point Likert-type rating scale. Regarding priorities of skills to be evaluated, we asked respondents to select up to 3 items from a 9-item list.

Responses were analyzed using the statistical package JMP 6.0.3 (SAS Institute Inc., Cary, NC, USA). The data were categorized into 2 main groups, and the Pearson Chi-square analysis was used to test statistical differences in proportions or distributions of nominal data. We considered a *p*-value of < 0.05 to indicate a statistically significant difference. The Tsukuba University Ethics Committee approved this research protocol prior to any data collection.

## Results

Of the 7,213 people who were randomly selected to participate in the survey, 3,532 people accessed the survey webpage and 3,093 (1,531 men and 1,562 women) completed the questionnaire (completion rate 42.9%). There were no significant difference between this respondents and Japanese population in age distribution and sex ratio.

The most requested skills for novice physicians were “explaining and discussing medical issues in an appropriate manner to patients” (n = 2,176; 70.4% of respondents), followed by “the skill of accurately diagnosing problems by physical examinations” (n = 1,984; 64.1% of respondents), and “carefully interviewing patients to make a diagnosis” (n = 1,663; 53.8% of respondents) [3] (Table 1).

**Table 1:** Survey results

**Factors which should be tested in medical licensure examinations**	**Respondents**	
	**(n = 3,093)**	
	**n**	**%**
Explaining and discussing medical issues in an appropriate manner to patients [2]	2176	70.4
Accurately diagnosing problems by physical examinations [3]	1984	64.1
Carefully interviewing patients to make a diagnosis [3]	1663	53.8
Reporting patients’ important problems correctly to their attending physicians, and having useful discussions about patients with their attending physicians	904	29.2
Skills of basic ambulatory care	803	26.0
Basic medical technique skills (i.e., intravenous injection, taking blood samples)	722	23.3
Being courteous	487	15.7
Recording medical documents accurately	320	10.3
Others	67	2.2

Otaki J et al. [3].

The table shows the factors respondents indicated as being necessary for the medical licensure examinations, followed by the numbers and percentage of respondents.

Overall, 90.5% of respondents (n = 2,800) answered that a national medical licensure examination must include a clinical skills test. On an univariate analysis, no statistically significant differences between this preference and respondents’ characteristics, such as sex, age, address, or medical history, were found. However, among the respondents who were skeptical toward medical care and health care utilization (n = 1,763; 57.0% of all respondents), this proportion was significantly higher (91.72%, *p* = 0.0092). Of all the respondents, 61.4% (n = 1,900) indicated that half or more of the cost should be borne by the government. Only 16.2% of all the respondents (n = 500) answered that medical students should be responsible for all costs.

## Discussion

We set out to clarify Japanese public opinion regarding the introduction of a clinical skills test into the national medical licensure examination. The present results revealed that the priority requirements with regard to clinical skills are not procedural skills such as basic medical emergency care or taking blood samples, but are communication skills, diagnostic interviewing and diagnostic physical examinations. This may contrast with some modern attitudes of physicians regarding excessive equipment-based care.

Medical school curricula have expanded significantly in accordance with advances in medical science and technology. Like other countries, Japanese medical education has been rapidly developing with introduction of modern communication trainings, skills labs, standardized patient encounters, small group teaching and nationwide core-curriculum [7]. However, the teaching of basic “interview and physical examination” skills needs to be developed simultaneously.

Nine of ten respondents answered in favor of adding a clinical skills test to the national medical licensure examination, especially those who were skeptical toward medical care and health care utilization. We should consider the difference between performance under test conditions and real life. Nevertheless, implementation of a clinical skills test may counteract such skepticism, because we can assure competency in novice physicians in some degree and increase trust in the medical system.

The substantial cost might be the biggest barrier to adding a clinical skills test. For example, the cost of standardized patients would be about US $2.5 million per year, if we introduced an objective structured clinical examination with 8 stations into the Japanese national medical licensure examination [8] Although most respondents in the present study answered that the cost should be borne by both the government and the medical students, the cost of a clinical skills test may nevertheless be an economic burden to the public.

The present study had several limitations. First, there was a possibility of selection bias because it was an internet-based survey. However, 79.3% of the public in 2006 [9] were internet users in Japan, and the ages of the present respondents were representative of the Japanese population. Second, our questionnaire did not provide any detailed information regarding the cost of a clinical skills test, because we could not obtain estimated data at that time, and thus there was a possibility that respondents could underestimate or overestimate the cost. Third, it might be difficult for respondents to discriminate among the requirements in a clinical skills test of a national medical licensure examination and the ideal competencies of physicians.

## Conclusion

The present study revealed a strong demand among the public to add a clinical skills test to the Japanese national medical licensure examination. Although the components of professional licensure examinations should depend not only on public demand but also on professional opinion, most of the respondents in this study strongly desired novice physicians to have a certain level of basic communication and physical examination skills. In addition to medical educators, medical doctors must also take the concerns of the public into consideration.

## Competing interests

The authors declare that they have no competing interests associated with this study.

## Authors’ contributions

JO conceived the study, contributed to the development of the design, received a research grant from the Grant-in-Aid for Scientific Research from the Ministry of Education, Culture, Sports, Science and Technology (MEXT), and contributed to the development of the questionnaire and the drafting of the manuscript. SNK performed the statistical analysis and contributed to the drafting of the manuscript. AT coordinated the research team, obtained the ethical approval and contributed in the development of the questionnaire. MO contributed to the development of the design, the questionnaire and the statistical analysis. MF contributed to the development of the design and the questionnaire. SM contributed to the development of the design, the questionnaire and the statistical analysis. All authors read and approved the final manuscript.

## Pre-publication history

The pre-publication history for this paper can be accessed here:

http://www.biomedcentral.com/1472-6920/13/74/prepub
